# Surgical complications of vagus nerve stimulation surgery: A 14-years single-center experience

**DOI:** 10.1016/j.bas.2023.102733

**Published:** 2023-12-14

**Authors:** Jouke van Schooten, Jacco Smeets, Sander MJ. van Kuijk, Sylvia Klinkenberg, Olaf E.M.G. Schijns, Jeske Nelissen, Louis G.L. Wagner, Rob P.W. Rouhl, Marian H.J.M. Majoie, Kim Rijkers

**Affiliations:** a Faculty of Health, Medicine and Life Sciences, Maastricht University, the Netherlands; b Department of Neurosurgery, Maastricht University Medical Center, the Netherlands; c School for Mental Health & Neuroscience, Maastricht University, the Netherlands; d Department of Neurology, Maastricht University Medical Center, the Netherlands; e ACE: Academic Center for Epileptology, Maastricht University Medical Center and Kempenhaeghe, Maastricht, Heeze, the Netherlands; f Department of Clinical Epidemiology and Medical Technology Assessment, Maastricht University Medical Center, the Netherlands

**Keywords:** Vagus nerve stimulation, Epilepsy, Surgical complications

## Abstract

**Introduction:**

Vagus nerve stimulation (VNS) is the most frequently used neuromodulation treatment for Drug-Resistant Epilepsy (DRE) patients. Complications of VNS surgery include surgical site infection and unilateral vocal cord paresis. Complication rates vary across studies.

**Research question:**

What is the safety profile of VNS related surgeries?

**Materials and methods:**

Retrospective cohort study using patient files of DRE-patients who had undergone primary implantation of a VNS-system, replacement of the VNS pulse generator, replacement of the lead, replacement of both pulse generator and lead, or VNS removal surgery in the Maastricht UMC+. Multiple Imputation was used for missing data. Univariable and multivariable logistic regression analysis were performed to analyze possible risk factors, in case of a small sample size, an independent-samples *t*-test and Fisher's exact test or Pearson's X^2^-test were used. The complication rate was calculated as percentage.

**Results:**

This study included a total of 606 VNS surgical procedures, leading to 67 complications of which 3 permanent complications. Complication rate after primary implantation was 13.4%; 2,5% for pulse generator replacement; 21.4% for lead revision and 27.3% for complete VNS removal. No statistically significant results were found when analyzing the results of adults and children <18 years separately.

**Discussion and conclusion:**

Complication rates of VNS-related surgeries in our own institutional series are low and comparable to previously reported series. VNS surgery is a relatively safe procedure. The complication rate differs per type of surgery and mean surgery duration was longer for patients with complications after lead revision surgery compared to patients without complications.

## Highlights

• The surgical complication rate of vagal nerve stimulation surgery performed in the MUMC+ from 2008 to 2022 is 11.1%.
• Lead removal or replacement surgeries are associated with a higher surgical complication rate than other VNS surgeries.
• Longer surgery duration is a possible risk factor for having a surgical complication after VNS lead revision surgery.

## 1 Introduction

Vagus nerve stimulation (VNS) is the most frequently used method of neuromodulation in patients with epilepsy. Between 1997 and 2021, more than 130.000 patients have been treated with VNS (Grasl et al., 2021; EpilepsieNL). The aim of VNS is seizure reduction; more than 50% seizure frequency reduction, is achieved in 35.7% of cases (Uthman et al., 1993). In addition to being clinically effective, VNS is cost-effective as well (Boon et al., 1999; Marras et al., 2020).

The VNS system consists of a pulse generator implanted below the left clavicle connected to a bipolar lead that is wrapped around the left vagus nerve in the sheath between the carotid artery and the jugular vein (Gonzalez et al., 2019). Although cardiac side-effects such as bradycardia or asystole as a result of VNS are very rare, they can occur. Usually, they are detected when testing the system during implantation, but few cases are known of patients having cardiac side-effects during VNS-therapy. Traditionally, the VNS-lead is wrapped around the left vagus nerve, because of the presumed increased risk of stimulation-induced cardiac side-effects with right-sided stimulation (Cantarin-Extremera et al., 2016). The exact mechanism of action of VNS therapy for drug-resistant epilepsy is unknown. The VNS-system will deliver electrical impulses to the vagus nerve, modulating neural circuits thought to be involved in seizure generation and propagation. It is proposed that VNS works by affecting cortical activity through projections of nucleus tractus solitarii to other nuclei in the brainstem, which are linked to different parts of the cortex. VNS possibly has an anti-epileptic effect by decreasing interictal events and by desynchronizing the activity of the cortex (Gonzalez et al., 2019).

The battery of the pulse generator has an average life span of 5–6 years depending largely on the daily use (Couch et al., 2016). When the end of battery life is near, renewal of the pulse generator is indicated. In some cases, lead replacement is required, because of lead damage due to wear or external forces (Aalbers et al., 2015; Spuck et al., 2010; Giordano et al., 2017). Some patients who do not benefit or experience adverse events from VNS request for removal of the VNS-system (Giordano et al., 2017). Removal either consists of complete removal, or of a tailored procedure where 2 cm of the lead (including the electrode coils wrapped around the nerve) are left in place.

Well-known complications that require adjustment of the treatment of VNS surgery include intraoperative asystole during lead impedance testing, damage to the thoracic duct, peritracheal hematoma, surgical site infection, and symptoms related to traction on the inferior recurrent laryngeal nerve including hoarseness, dyspnea, and dysphagia (Giordano et al., 2017).

Some complications do not require treatment adjustment and do not lead to irreversible damage to the patient. These complications include jugular vein injury that is managed (stitched) during the same procedure or wound hematoma around the pulse generator.

Complication rates range between 4.2 and 16.7%, depending on the exact definition of a surgical complication and the type of VNS surgery evaluated (primary implantation, pulse generator replacement, lead replacement, or removal surgery) (Lotan and Vaiman, 2015; Révész and Ben-Menachem, 2016; Kahlow and Olivecrona, 2013; Spindler et al., 2021). Well-known risk factors for getting surgical complications in general include smoking, obesity, diabetes, duration of surgery, use of immunosuppressive medication, and length of preoperative hospitalization (Anderson, 2011). Intellectual disability is sometimes considered a risk factor because of drooling or wound care management (Spuck et al., 2010; Le et al., 2002).

Studies that describe all types of VNS surgeries are sparse and to our knowledge, no other studies have investigated specific risk factors for VNS surgeries before. Therefore, the purpose of this single-center retrospective cohort study was to assess surgical complication rates of all types of VNS surgeries performed in the Maastricht MUMC + between 2008 and 2022 and to investigate potential risk factors for getting a surgical complication. As such, this is the first large study that investigates all types of VNS surgeries in substantial numbers.

## 2 Materials and methods

### 2.1 METC approval

Due to the retrospective nature of the study, the medical ethics committee of MUMC + decided that this study does not fall under the Medical Research Involving Human Subjects Act (WMO, number: METC, 2022-3104-A-3).

### 2.2 Data collection

The electronic patient files were checked based on surgical codes to find all patients who underwent VNS surgery between January 2008 and October 2022. Patient data extracted from the system included sex, age at surgery, comorbidities including diabetes and intellectual disability, smoking habits, Body Mass Index (BMI), date of surgery, number and dates of subsequent VNS surgeries, surgery duration, timing of antibiotics application prior to surgery and the occurrence of surgical complications. To store the collected data, an online research database (Castor Electronic Data Capture, v2023.2.1.0) was used.

### 2.3 Surgical procedures; general information

All surgeries were performed by neurosurgeons of the Maastricht University Medical Centre (MUMC+) and took place under general anesthesia. The surgical field was disinfected with chlorhexidine. The procedure was performed under strict sterile conditions with, amongst others, the use of double hand gloves and no opening of the operation room (OR) doors during the implantation. All patients left the hospital one day after surgery if recovery was uneventful.

### 2.4 General description of surgical procedures

#### 2.4.1 Primary implantation surgery

Two incisions are required: one left-sided lower cervical incision, one in the left infraclavicular area. First, the cervical incision is made, followed by atraumatically preparing a corridor on the mesial side of the sternocleidomastoid muscle to the vascular-nerve sheath after which the vagus nerve is identified between the internal jugular vein and carotid artery. The nerve is exposed for approximately 3 cm. Next, the infraclavicular incision is made, and the pocket is prepared either subcutaneously or subpectorally. The packaging of the system is opened, the lead tunnel is prepared, the lead is placed in this subcutaneous tunnel and the electrode coils are placed around the vagus nerve with aid of a microscope or magnifying glasses. The lead is fixed with a tension-relief loop using two anchors attached to the hypopharynx mesially and sternocleidomastoid fascia laterally. Next, the lead is connected to the pulse generator, which is placed in the subcutaneous or subpectoral pocket. Subsequently, lead impedance is determined and potential stimulation-induced changes in heart rate are noted. Additionally, the incision sites are flushed with several milliliters of a 20-mg/mL gentamycin solution and closed in layers, finishing off with intracutaneous stitching. Surgery is performed under 24 h of prophylactic antibiotics (cephazolin, 2 g prior to surgery followed by 1 g every 6 h for three doses) or clindamycin (600 mg prior to surgery followed by 600 mg every 6 h in three doses) in case of cephazolin allergy. In children, weight is used to calculate the appropriate doses.

#### 2.4.2 Pulse generator replacement

For pulse generator replacement, the infraclavicular incision is opened. Next, the generator is gently moved out of the pocket. The lead is disconnected. A new generator is plugged into the lead and is repositioned inside the pocket after which lead impedance is determined and the generator programmed with the requested stimulation parameters. Finally, the wound is irrigated with several milliliters of a 20-mg/mL gentamycin solution and closed in layers, finishing off with intracutaneous stitching.

#### 2.4.3 Complete revision surgery or complete removal surgery

The surgical procedure of VNS complete revision or removal surgery has been described in detail earlier (Aalbers et al., 2015). In order to perform complete revision or removal of the VNS-system, the generator is removed from the pocket and the lead is disconnected. Then the cervical incision is reopened to expose the lead. For this, the fibrotic tissue is dissected carefully to find the anatomic landmarks of the carotid artery and jugular vein. After lead exposure, the fixation booklets are removed, and the lead is exposed by carefully opening the scar tissue with the aid of a microscope. In case of complete revision surgery, a new electrode and pulse generator are placed in a way similar to the primary implantation procedure (Kahlow and Olivecrona, 2013).

### 2.5 Complication definition

In this study, a complication is defined as an unintended effect caused by surgery. Examples of events considered as complications in this study are: infections, wound hematomas, injuries to surrounding tissues (the jugular vein, vagal nerve or thoracic duct), damage to hardware caused by the surgery and symptoms related to traction on or manipulation of the recurrent laryngeal nerve (transient hoarseness of which patients normally recover within a few months, and permanent hoarseness). (Giordano et al., 2017).

### 2.6 Statistical analysis

Statistical analysis was carried out using IBM SPSS version 28 (IBM Corp, Armonk, NY). Patient characteristics were analyzed and summarized using descriptive statistics. Continuous variables were, unless otherwise indicated, presented as mean with standard deviation (SD), and categorical variables were presented as a number with a percentage.

Multiple imputation was performed to impute incomplete variables. The assumption of Missing At Random (MAR) was made for the missing data. We set the number of imputations to 5 and used predictive mean matching to draw values for continuous variables. Complication rates were calculated by dividing the number of complications by the number of surgeries. Next, univariable and multivariable logistic regression analysis were performed to analyze possible risk factors for complications. In case the number of events was too small for the logistic regression model to estimate associations, we used the independent-samples *t*-test to compare means between those with and without complications and Fisher's exact test or Pearson's X^2^-test to compare proportions between groups. A p-value of <0.05 was considered to be statistically significant.

## 3 Results

### 3.1 Demographics

In total, 606 VNS procedures were performed in the MUMC+ from 2008 to 2022 in 437 patients, consisting of either primary implantation of a VNS system (n = 306), replacement of the VNS pulse generator (n = 201), replacement of the lead (n = 42), VNS removal surgery (either complete removal (n = 44) or incomplete removal (n = 3), or any other VNS-related surgery (n = 10). Surgery-related complications occurred in 67 surgeries and consisted of infection (n = 14), damage to the thoracic duct (n = 1), vocal cord paresis either permanent (n = 1) or transient (n = 5), or vocal cord paresis loss to follow up (n = 8), hoarseness without confirmed vocal cord paresis (n = 11), damage to the lead during surgery (n = 1), high impedance during testing one day after surgery (n = 2), opening the wrong incision (n = 1), severe damage to the vagus nerve during surgery (n = 1), jugular vein injury (n = 5), wound hematoma (n = 4), allergic reaction to iodine/chlorhexidine, sterile drapes or plasters and medication used during surgery (n = 5), mild and temporary throat symptoms attributed to traction on the nerve (n = 4), mechanical or technical problems (n = 5). The different types of complications that occurred are also shown in Table 2.

### 3.2 Primary implantation

Primary implantation was performed in 306 patients, see Table 1 for patient characteristics. In total, 41 complications occurred, consisting of wound infection (n = 11), hoarseness (n = 16), of which one permanent vocal cord paresis (n = 1), thoracic duct leakage (n = 1), high impedance during testing one day after surgery (n = 2), wound hematoma (n = 2), allergic reaction to iodine/chlorhexidine, sterile drapes or plasters and medication used during surgery (n = 5), mild and temporary throat symptoms attributed to traction on the nerve (n = 4).

**Table 1:** Patient characteristics by procedure.

	Primary implantation (n = 306)	Generator replacement (n = 201)	Complete revision (n = 42)	Removal (full) (n = 44)	Removal (partial) (n = 3)
Women (%)	151 (49.3)	98 (48.8)	18 (42.9)	23 (52.3)	1 (33.3)
Children age<18 (%)	76 (24.8)	32 (15.9)	13 (31.0)	10 (22.7)	0
BMI at implantation (Mean in kg/m^2^ (SD))	24.05 (5.20)	24.8 (5.73)	23.59 (6.80)	23.52 (5.15)	27.59 (0.229)
Age at surgery (Mean in years (SD))	30.86 (16.94)	34.11 (15.36)	29.07 (16.74)	30.93 (14.44)	41 (12.17)
Surgery duration (Mean in minutes (SD))	83.36 (23.38)	38.9 (16.34)	128.12 (33.29)	78.18 (21.34)	82.5 (9.19)
N Smokers (%)	45 (14.7)	16 (8.0)	5 (11.9)	6 (13.6)	0
N Diabetes (%)	4 (1.3)	1 (0.5)	0	2 (4.5)	0

**Table 2:** Surgical complication rate of each type of complication for primary implantation, lead revision surgery, and generator replacement surgery separately. Complications occurred in the primary implantation group, generator replacement group, lead revision group and full removal surgery group. The other types of surgeries are not shown in this table.

Complication	Primary implantation (n = 306)	Generator replacement (n = 201)	VNS-system revision (n = 42)	VNS full removal (n = 44)
Infection (%)	11 (3.6)	0	1 (2.4)	2 (4.5)
Hoarseness (%)	16 (5.2)	0	4 (9.5)	5 (11.4)
From which vocal cord paresis, concluded by ENT doctor:				
• Transient	1 (0.3)		1 (2.4)	3 (6.8)
• Permanent	1 (0.3)		0	0
• Loss to follow up	3 (1.0)		3 (7.1)	2 (4.5)
Jugular vein injury (%)	0	0	3 (7.1)	2 (4.5)
Hematoma (%)	2 (0.7)	1 (0.5)	0	1 (2.3)
Other (%)	12 (3.9)	4 (2.0)	1 (2.4)	2 (4.5)
Total (%)	41 (13.4)	5 (2.5)	9 (21.4)	12 (27.3)

Management of infection consisted of antibiotics in all cases (up to 3 months). Four of the infected cases required additional surgery, in two patients this consisted of wound revision surgery, and in two patients the complete VNS-system needed to be removed in order to effectively treat the infection.

The patient suffering from thoracic duct leakage was treated with a fat-free diet for 7 days. In the two patients with high impedance, surgical re-exploration of the pulse generator-lead connection was carried out. Indeed, in these cases, the lead was not properly connected to the pulse generator during implantation surgery. None of the above-described complications were permanent, even though for the two patients in whom the complete VNS-system was removed in order to control the postoperative infection, it meant that they were not able to receive VNS treatment anymore. Details about the types of complications are shown in Table 2.

### 3.3 Safety analysis for complication after primary implantation

Univariable and multivariable logistic regression analysis revealed that none of the potential risk factors showed a statistically or clinically relevant association with the occurrence of a complication. Fisher's exact test revealed no association between intellectual disability and the incidence of an infection; 5/107 patients with an intellectual disability presented with an infection compared to 6/199 patients without an intellectual disability, p = 0.329.

### 3.4 Pulse generator replacement

Pulse generator replacement was performed in 201 patients, see Table 1 for patient characteristics. Five complications occurred in this series, which consisted of vasovagal symptoms, such as lightheadedness, feeling warm of experiencing cold, clammy sweat (n = 1), unusual amount of pain (n = 1), wound hematoma (n = 1) and hypesthesia of the scar tissue (n = 1). The fifth complication consisted of opening of the wrong incision (n = 1). Primary implantation of this patient had taken place in another hospital where the subcutaneous pocket had been placed on the left shoulder. The reason for that location was, that the patient already had a subclavian line in the left infraclavicular area during primary implantation surgery. During pulse generator replacement surgery six years later, the subclavian line was not present anymore, and the subclavian line scar was misinterpreted for the VNS pulse generator scar. This ‘wrong’ incision was closed, and the right incision was opened to replace the pulse generator. The complication did occur in a mentally disabled patient in whom the normal time out procedure, where the patient is asked to confirm the site of incision, was not carried out properly.

### 3.5 Safety analysis for pulse generator replacement

Because only five complications occurred, no formal statistical analysis was performed.

### 3.6 Revision of the VNS-system

In total 42 complete revisions were performed, see Table 1 for patient characteristics. Complications occurred in 9 cases and consisted of hoarseness (n = 4), surgical site infection (n = 1), surgical damage to a newly implanted silicone cover of the lead during surgery (n = 1) and jugular vein injury (n = 3). The surgical site infection was treated with antibiotics for 3 months. When surgical damage of the silicone cover of the lead was discovered, the lead was revised during the same procedure.

### 3.7 Safety analysis for complete revision

Mean surgery duration was 150,71 min for patients with surgical complications compared to 123,64 min for patients without surgical complications, p = 0.039. None of the other potential risk factors showed a statistically or clinically relevant association with the occurrence of a complication.

### 3.8 *VNS* removal

VNS removal surgeries were performed in 47 patients. Forty-four patients underwent full removal of the complete system including electrodes. In the other three patients, the proximal part (2–3 cm) of the lead with electrode coils was left around the nerve. VNS removal was performed in most cases because of ineffectiveness (n = 30) or stimulation related side effects (n = 12). Two patients received VNS removal because of an ongoing infection. Three patients were cured from their seizures as a result of other treatments.

Patient characteristics are shown in Table 1 for both the full and partial removal surgery. Complications occurred in 12 patients, all of them occurred in the full removal group, and consisted of infection (n = 2), hoarseness (n = 5), visibly damaged vagus nerve (n = 1), jugular vein injury (n = 2), wound hematoma (n = 1), and dysphagia (n = 1).

### 3.9 Safety analysis for VNS partial removal

No complications occurred during these surgeries. Therefore, no complication incidence analysis was performed.

### 3.10 Safety analysis for VNS full removal

In the 44 full removal cases, no statistically or clinically significant differences were found between patients with surgical complications compared to patients without surgical complications.

### 3.11 Other surgeries

Other surgeries consisted of wound revisions (n = 2) or pulse generator repositioning (n = 8). No complications occurred during these surgeries.

### 3.12 Adults versus children

All of the analyses described above were also done in only adults and only children below 18 years of age. None of the above variables were statistically significant when only looking at adults or children.

## 4 Discussion and conclusion

### 4.1 Discussion

This study aimed to describe the surgical complication rate of VNS implantation, pulse generator replacement, lead revision, and VNS removal surgery, and assess risk factors. This single-center study evaluated a cohort of 606 surgeries that were performed in the MUMC+ from January 2008 to October 2022. Of these 606 surgeries, 306 were primary implantations, 201 were pulse generator revisions, 42 were complete revisions, 44 were complete removal surgeries and 3 were the partial removal of the pulse generator and the lead leaving 2–3 cm behind including the electrode coils surrounding the nerve. The other 10 surgeries consisted of both wound revisions and pulse generator repositioning. In this study, the types of complications differ per type of surgery. Surgical complication rate for primary VNS implantation in this study was 13.4%. In the pulse generator replacement cohort, 5 complications occurred in 201 surgeries. In the revision cohort 9 complications occurred in 42 surgeries. This study found a total surgical complication rate of 11.1%. The complication rate of this study corresponds to previous literature that showed surgical complication rates ranging from 4.2 to 16.7%. (Lotan and Vaiman, 2015; Révész and Ben-Menachem, 2016; Kahlow and Olivecrona, 2013; Spindler et al., 2021). In total, three permanent complications occurred. Therefore, the complication rate was 0.5% for permanent complications: 1 permanent vocal cord palsy and 2 patients who needed removal after infection. In this study, no cardiac side effects did occur.

Complications of VNS surgery are seldom life-threatening but can impair the patient's quality of life. Surgical site infection can lead to VNS removal. After surgical site infection requiring VNS removal, patients will never receive a new left-sided VNS-system again. Right-sided VNS implantation is not performed in The Netherlands, because of the once presumed risk of cardiac side effects and greater experience with left-sided VNS considering effectiveness and complications. We did not include lead-replacement and VNS removal as complications, because we considered this to be a complication of the VNS-system, instead of being a complication from the patient's body or as a result of surgical actions. Because VNS-therapy is relatively new, we don't know yet how many years we should expect a lead to stay in place.

Apart from its seizure suppressing effect, VNS is associated with a positive influence on memory, concentration, and mood. Therefore, removal in case of infection permanently prevents these patients of the chance of benefiting from VNS.

Another surgical complication, influencing quality of life, is permanent failure of the recurrent laryngeal nerve, which can lead to left-sided vocal cord paresis and consequently complaints of dyspnea, cough, and hoarseness; this only occurred once in this series. In this particular patient, VNS has a good seizure-suppressing effect, making the occurrence of this complication acceptable for the patient.

This study shows a high complication rate for complete removal surgery. Complete removal of the VNS-system is generally not necessary. For example, a patient is still allowed to undergo an MRI if the distal 2–3 cms of the lead including the electrode coils are left in place. Based on the results of this study and especially in the light of the complication risk, we advise to reconsider each request for complete removal and discuss the best strategy in a multidisciplinary setting of the epilepsy surgery conference.

### 4.2 Safety analysis

One statistically significant risk factor has been found in this study. Mean surgery duration was longer for patients with complications after lead revision (150.71 min) compared to patients without complication (123.64 min). The results of this study on surgery duration hold an important caveat, because it is unclear whether surgery duration is reported accurately. In daily practice, the end-of-surgery moment is reported by the anesthetics nurse who often reports a similar time point for end-of-surgery and end-of-anesthesia. Therefore, the surgery time might be overrated in our study.

In our cohort, the mean time of antibiotics administration prior to incision was less than 30 min. Previous literature shows that antibiotics should be given within 30–60 min before incision, which means that the antibiotics were administered relatively late in our cohort (MF and M, 2022; Bratzler and Houck, 2005).

### 4.3 Study limitations

The largest limitation of this study is the retrospective design, which led to missing data. Another limitation of this study is that no correction was used in the analysis of the generator replacement or lead revision surgeries. Possible bias occurred because some subjects were operated on multiple times and were included in the data collection and analysis multiple times. If a patient had a surgical complication at the first surgery, it was expected that the risk of having a surgical complication at the following surgery would be higher (Kahlow and Olivecrona, 2013). For this increased risk, no correction was applied. Another limitation of this study is the small sample size in the pulse generator, lead revision, and VNS removal surgery groups. This is why no formal statistical analysis could be performed for these groups. The logistic analysis for the primary implantation group was performed with a limited sample size as well. Because of this, the analysis of possible risk factors in this study has to be interpreted with care.

### 4.4 Strengths and future perspectives

This study is the first to our knowledge that studies a cohort of this size and compares the different types of surgeries. Most literature studies focus on only one type of surgery. For future studies, larger sample sizes are recommended to analyze possible risk factors for having a surgical complication. The epilepsy population consists of a relatively large number of intellectually disabled patients. Therefore, getting insight into the complications that occur in this specific population and possible risk factors might be relevant as well.

### 4.5 Conclusion

This single-center, retrospective study shows that VNS implantation is a relatively safe procedure, with a total complication rate of 11.1% complications in 606 surgeries of which 3 permanent complications. The most common complication was transient hoarseness, followed by surgical site infection. An association was noted between surgery duration and complications in the lead revision group. This study, unique in its analysis of different types of surgeries, demonstrated that VNS removal or lead revision surgery might be related to a higher complication rate compared to generator replacement or primary implantation. More research, both on adults and children, is needed to confirm these results.

## Contributors

Jouke van Schooten: planning and designing the study, writing the study protocol, retrieving data from patient charts.

Jacco Smeets: retrieving data from patient charts, reviewing the protocol and editing the manuscript.

Sander van Kuijk: giving advice on methodology, reviewing the protocol and editing the manuscript.

Sylvia Klinkenberg: reviewing the protocol and editing the manuscript.

Olaf Schijns: reviewing the protocol and editing the manuscript.

Jeske Nelissen: reviewing the protocol and editing the manuscript.

Louis Wagner: reviewing the protocol and editing the manuscript.

Rob Rouhl: reviewing the protocol and editing the manuscript.

Marian Majoie gave critical feedback on the article and edited the manuscript.

Kim Rijkers: planning and designing the study, writing the study protocol, reviewing the protocol and editing the manuscript.

## Funding

This research did not receive any specific grant from funding agencies in the public, commercial, or not-for-profit sectors.

## Declaration of competing interest

None declared.

## Figures and Tables

**Table 3:** percentage of missing data per variable with missing data. The assumption of Missing At Random (MAR) was made for the missing data.

	Primary implantation	Pulse generator replacement	Lead replacement	Removal surgery
Duration of antibiotics	35.3%	15.5%	25.6%	38.3%
Surgery duration	22.5%	4.5%	2.3%	12.8%
BMI	15.4%	4.5%	7.0%	10.6%
Smoking	3.3%	0	0	8.5%
Diabetes	2.0%	0	0	4.3%
